# Monitoring Study Participants and Implementation with Phone Calls to Support Hypertension Control During the COVID-19 Pandemic: The Case of a Multicomponent Intervention Trial in Guatemala

**DOI:** 10.5334/gh.954

**Published:** 2021-11-24

**Authors:** Diego Hernández-Galdamez, Kristyne Mansilla, Ana Lucía Peralta, Javier Rodríguez-Szaszdi, Juan Manuel Ramírez, Dina Roche, Pablo Gulayin, Manuel Ramirez-Zea, Jiang He, Vilma Irazola, Meredith P. Fort

**Affiliations:** 1 INCAP Research Center for the Prevention of Chronic Diseases (CIIPEC), Institute of Nutrition of Central America and Panama (INCAP), Guatemala City, GT; 2 Institute for Clinical Effectiveness and Health Policy (IECS), Buenos Aires, AR; 3 Tulane University School of Public Health and Tropical Medicine, New Orleans, Louisiana, US; 4 Colorado School of Public Health, Anschutz Medical Campus, Aurora, Colorado, US

**Keywords:** Hypertension, Non-communicable Diseases, Covid-19, mHealth, Cardiovascular Diseases, Implementation Science

## Abstract

**Background:**

The COVID-19 pandemic presents a challenge to health care for patients with chronic diseases, especially hypertension, because of the important association and increased risk of these patients with a severe presentation of COVID-19 disease. The Guatemalan Ministry of Health has been implementing a multi-component program aimed at improving hypertension control in rural communities since 2019 as a part of an intervention research cluster randomized trial. When the first cases of COVID-19 were reported (March 13, 2020) in Guatemala, our study paused all study field activities, and began monitoring participants through phone calls. The objective of this paper is to describe the approach used to monitor study participants during the COVID-19 pandemic and compare data obtained during phone calls for intervention and control group participants.

**Methods:**

We developed a cross-sectional study within the HyTREC (Hypertension Outcomes for T4 Research within Lower Middle-Income Countries) project ‘Multicomponent Intervention to Improve Hypertension Control in Central America: Guatemala’ in which phone calls were made to participants from both intervention and control groups to monitor measures important to the study: delivery of antihypertensive medications in both groups, receipt of coaching sessions and use of a home blood pressure monitor by intervention group participants, as well as reasons that they were not implemented.

**Results:**

Regarding the delivery of antihypertensive drugs by the MoH to participants, those in the intervention group had a higher level of medication delivery (73%) than the control group (51%), p<0.001. Of the total participants in the intervention group, 62% had received at least one health coaching session in the previous three months and 81% used a digital home blood pressure monitor at least twice a week. Intervention activities were lower than expected due to restricted public transportation on top of decreased availability of health providers.

**Conclusion:**

In Guatemala, specifically in rural settings, access to antihypertensive medications and health services during pandemic times was impaired and less than expected, even after accounting for the program’s implementation activities and actions.

## Introduction

Since December 2019, more than 188 countries have been affected by the COVID-19 pandemic [1]. This acute respiratory syndrome caused by the virus SARS-CoV-2 binds its viral surface spike protein to the human angiotensin-converting enzyme 2 (ACE2) receptor [2]. Thus, patients with previous cardiovascular conditions who remain in states of excessive activation of the renin-angiotensin system have increased morbidity and mortality from COVID-19. In addition to the risk of severe disease and death caused by the affinity of the virus to ACE2 receptors, patients with cardiovascular diseases also have an underlying pro-inflammatory state and decreased immune activity, which enhances COVID-19 severity [2]. Cardiovascular complications due to COVID 19 infection include myocardial injury, cardiac arrhythmias, pulmonary or systemic thromboembolism, and decompensated cardiac failure [3].

The COVID-19 pandemic presents a challenge to health care for patients with non-communicable diseases -NCDs. Hypertension, is a particular concern, because of the important association and increased risk of these patients with a severe presentation of COVID-19 disease [4,5]. In Guatemala, the Ministry of Health (MoH) recommended in its guidelines the suspension of outpatient consultations in health services and delivery of medicine through third parties so that patients reduced their risk of exposure [6]. As it was initially unclear what the institutional response would be for hypertensive patients, our study team considered that it would be important to reach out to and monitor study participants. Control of NCDs, such as hypertension, in low- and middle-income countries (LMICs) before the pandemic was challenging and is now notably worse. Considering that hypertensive patients, and those with NCDs, are more vulnerable to COVID-19, the management of these diseases must be prioritized and adapted during this pandemic [7].

A rapid assessment of service delivery for NCDs during the COVID-19 pandemic among 163 Ministries of Health (MoH) revealed that 75% (122 countries) reported NCDs services being partially or completely disrupted; a situation that worsened with the severity of the transmission phase in each setting. Some causes of service disruption that have been described include: cancellation of elective care (in 65% of the countries), government or public transportation lockdowns (43%), closure of outpatient disease clinics (34%), insufficient staff to provide health care (33%), patients not showing up at clinics (25%), and unavailability of essential medicine (20%) [8]. NCD staff members in 94% of the MOHs assessed were reassigned to help with the COVID-19 response. Fifty percent of countries reported that screening programs were also postponed since WHO’s initial recommendation was to minimize non-urgent facility-based activities. According to WHO, disruption in service delivery is a global issue; however, the problem is worse in LMICs [9].

Guatemala confirmed its first COVID-19 case on March 13, 2020, which was followed by the early implementation of a nationwide plan for prevention, containment, and response to the epidemic [10]. Although quick action was taken by the government and the MoH, by August 21st the country had 66,941 reported cases and 2,532 deaths [11]. Several factors such as service delivery and the population’s vulnerability challenged the country’s response and control of the disease; for instance, Guatemala has a total of 60,475 health professionals – including physicians, professional and auxiliary nurses, health facilitators, and others – and a network of 1,545 service units to provide health care to the communities [10]. Data describing Guatemala’s 2017 epidemiologic profile stated that prevalence rates for hypertension and diabetes mellitus were 764 and 579 per 100,000 habitants respectively, evidencing the vulnerability of the population to COVID-19 and the urgency to intervene [12].

Since 2019, we have been implementing a study titled ‘Implementation of a multicomponent intervention to improve hypertension control in Central America, stage 1: Guatemala.’ The study is a type 2 hybrid effectiveness-implementation cluster-randomized trial that aims to evaluate whether a multi-component program that works at the patient, provider, and system levels is effective in improving hypertension control in adults who receive care at MoH centers and posts in rural Guatemala. The study includes 36 health districts, 18 randomized to the multicomponent intervention, and 18 randomized to the usual care group within 5 health areas of the country (Baja Verapaz, Chiquimula, Huehuetenango, Solola, and Zacapa).

The study intervention is part of the HyTREC/TREIN, NHLBI-funded consortium of projects that aims to increase capacity in LMICs to design, implement, and assess interventions that address the cardiovascular disease burden globally and to improve the global health community’s understanding of the barriers and opportunities specific to LMICs and the strategies best suited for low-resource settings. The study is a multicomponent and multilevel program implemented within the first and second levels of care, at health posts and health centers of the Guatemalan MoH. The program is composed of one core intervention (protocol-based treatment) and five evidence-based implementation strategies (team-based collaborative care, health provider training, health coaching sessions, home blood pressure monitoring, and blood pressure audit and feedback). MoH physicians and nurses working at intervention health centers and auxiliary nurses working at health posts are responsible for delivering the intervention. The control group receives enhanced usual care since the study provided an electronic blood pressure monitor to the participating health posts and centers and works with MoH officials to promote the purchase, distribution, and availability of essential hypertensive medications [13].

When the first cases of the COVID-19 pandemic started in Guatemala, due to government restrictions, our study paused all field activities related to participants’ follow-up by field evaluators (such as blood pressure and weight measurement, and application of questionnaires about lifestyle, medication adherence, behavior change, etc.) that measure the intervention’s effectiveness and participants’ receipt of the intervention. In addition, while COVID-19 cases increased nationally, local governments at the study sites established mobility restrictions, *cordons sanitaire*, and curfews that partially disrupted service delivery at study sites. Therefore, hypertensive participants enrolled in the study were not receiving healthcare as usual. In response, diverse strategies have been recommended to mitigate and ensure the continuity of service delivery for people living with NCDs, including prioritization of services for the major NCDs, telemedicine, novel dispensing approaches including multi-month medication refills, among others [12,14]. This paper aims to describe the approach that we used to monitor study participants during the COVID-19 pandemic and compare delivery of medications for intervention and control group participants, and document receipt of coaching sessions, use of blood pressure monitors, and reasons that these were not implemented for those in the intervention group.

## Methods

We conducted a cross-sectional analysis within the HyTREC project ‘Multicomponent Intervention to Improve Hypertension Control in Central America: Guatemala,’ to monitor participants, understand the implementation of two of the program’s strategies and capture how the response to the COVID-19 pandemic emergency influenced hypertension care for those enrolled in the study.

### Participants

By March 2020, 1,402 participants across 36 health districts had been recruited. Due to the suspension of study activities, recruitment was paused in March. A total of 1,384 active participants continued in the study as of August 2020, and recruitment was completed after restrictions were lifted.

### Materials

The study team developed a monitoring system in the form of monthly telephone calls to every study participant to understand the study’s status within the five health areas. For this, a standardized 7-minute questionnaire (for both intervention and control group participants) and a 15-minute standardized script (for the intervention group only) were developed by the research team. Both instruments focused on the participant’s status and the implementation of the intervention activities:

*Questionnaire*: Consisted of four items that assessed anti-hypertensive medications for study participants, including aspects such as medication delivery by the MoH, location of medication delivery, and medication adherence. This questionnaire was answered by participants in the intervention and control groups. Six additional items regarding participation in health coaching sessions, location where the sessions were received, and the use of a home blood pressure monitor were also included in the intervention group questionnaire. Reasons that coaching sessions were not offered and that the blood pressure monitor was not used were documented and summarized.

*Script*: This consisted of standardized information that was delivered to the participants of the intervention group only, where data collectors provided healthy lifestyle recommendations (physical activity, DASH diet) and tips for improving medication adherence to antihypertensive medications. Additionally, the calls included reminders to attend health coaching sessions and to use a home blood pressure monitor and document measurements.

Materials were translated orally into five Mayan languages (Achí, K’iche’, Kaqchikel, Tz’utujil, and Mam), since at least 39% of enrolled participants speak only one of these languages or are not completely fluent in Spanish.

### Variables

From the questionnaire applied during the phone call, information was obtained on:

Intervention and control groups:

*Antihypertensive medications*: 1) delivery by MoH in the last month; 2) location of medication delivery; 3) actions taken if they did not receive medication, and 4) adherence.

Intervention group only: *Health coaching sessions*: 1) Participation in at least one health coaching session in the previous three months, 2) the location where the health coaching session took place, and 3) reasons for not having received the health coaching session.

*Blood pressure monitor*: 1) Proper use of the digital blood pressure monitor at home in the last month (2 times per day, twice a week), and 2) reasons for not using the digital monitor.

### Training

Field evaluators are certified study personnel responsible for recruitment of and home-based visits to each participant, in both intervention and control groups. These evaluators were hired for the study and are not MoH staff, and do not deliver the intervention but rather collect data to assess program effectiveness and the extent to which intervention group participants receive the intervention. In early March, theses field evaluators were virtually trained in the appropriate use of the questionnaire and script over the phone, in Spanish and a Mayan language, if applicable.

### Implementation

Starting in March 2020, study participants who were enrolled in the trial were contacted via telephone calls. By June 2020, four telephone calls were made to the participants randomized in the intervention group, and two telephone calls to the participants in the control group. The last phone call took place in June and was made to both groups.

### Statistical analysis

Data were collected and managed using *REDCap* electronic data capture tools hosted at the Institute of Nutrition of Central American and Panama (INCAP) in Guatemala City.

We analyzed data from the June phone calls because they were made to both intervention and control group participants, and all the data gathered through this call was managed using *REDCap* electronic data capture tools hosted at INCAP. We ran a Mann-Whitney U test to determine differences between intervention and control groups for age. Categorical variables, like the variables contained in the main outcomes (antihypertensive medications, coaching sessions, and blood pressure monitor), were described as percentages. Chi-square test and Fisher’s exact test were performed to compare intervention and control group participants. All statistical analysis was performed using *Stata SE version 15.0 software (Stata Corporation, College Station, TX, USA)*.

## Results

Table 1 presents an overview of the characteristics of study participants who answered monitoring phone calls in June of 2020. Of the total of participants enrolled by the time phone calls were made, 94% of the intervention group and 89% of the control group answered the phone calls. The median age of participants was 63 years; more than 70% of participants were women, and more than 50% were illiterate. 71% of participants in the intervention group and 67% in the intervention group had a diagnosis of hypertension prior to the study. The most frequently reported comorbidities in the intervention and control groups included: overweight/obesity (24.4%, 17.5%), dyslipidemia (23.6%, 18.6%), diabetes mellitus (20.5%, 17%) and depression (20.4%, 16.4%). The only significant differences between groups were found in overweight/obesity and ethnicity variables.

**Table 1:** General characteristics of study participants who answered the phone call, June 2020.

	Total	Intervention	Control	p-value

**Total population**	1282	677	605	
**Participants who answered the phone call**				
**Age, median (IR)**	63 (54,71)	63 (54,71)	63 (54,70)	0.415
**Women**	919 (71.68%)	488 (72.08%)	431 (71.24%)	0.738
**Health area**				0.917
Baja Verapaz	165 (12.87%)	84 (12.41%)	81 (13.39%)	
Chiquimula	379 (29.56%)	197 (29.10%)	182 (30.08%)	
Huehuetenango	253 (19.73%)	139 (20.53%)	114 (18.84%)	
Sololá	399 (31.12%)	213 (31.46)	186 (30.74%)	
Zacapa	86 (6.71%)	44 (6.50%)	42 (6.94%)	
**Ethnic group**				0.013
Maya	542 (42.31%)	271 (40.09%)	271 (44.79%)	
No Maya	339 (26.46%)	202 (29.88%)	137 (22.64%)	
Do not know	400 (31.23%)	203 (30.03%)	197 (32.56%)	
**Literate**	553 (43.13%)	292 (43.32%)	261 (43.21%)	0.968
**Previous diagnosis of hypertension**	881 (68.88%)	478 (70.71%)	403 (66.61%)	0.114
**Medical history**				
Dyslipidemia	273 (21.29%)	160 (23.63%)	113 (18.68%)	0.091
Overweight/obesity	271 (21.14%)	165 (24.37%)	106 (17.52%)	0.008
Heart attack	58 (4.52%)	35 (5.17%)	23 (3.80%)	0.408
Cerebrovascular event	98 (7.6%)	61 (9.02%)	37 (6.12%)	0.148
Diabetes	242 (18.88%)	139 (20.53%)	103 (17.05%)	0.210
Depression	237 (18.49%)	138 (20.41%)	99 (16.36%)	0.174
Cancer	11 (0.86%)	9 (1.33%)	2 (0.33%)	0.106
COPD	59 (4.60%)	28 (4.14%)	31 (5.13%)	0.429

IR = Interquartile range, COPD = Chronic obstructive pulmonary disease.

Table 2 compares delivery of medications for participants in the intervention and control groups. Regarding the delivery of antihypertensive medications to participants by the MoH, a higher proportion was delivered to participants in the intervention group (73%) as compared to the control group (51%), p<0.001. No significant differences were found between groups as to the location of medication delivery. A greater proportion of participants in the control group had not received antihypertensive medications in the last month (46.51%) and had to buy medications (42%), compared to those in the intervention group (37.5% and 35%, respectively). In the intervention group, 86% of participants had enough medication for the next month, as compared to 69% of the control group, whether they received it in the health post, bought it on their own or had received enough medicine for more than one month. As for adherence, a greater proportion of participants in the intervention group reported taking the medication ‘always’ (80%) compared to the control group (65%), p < 0.001.

**Table 2:** Delivery and adherence to antihypertensive medications, June 2020.

	Total	Intervention	Control	P-value

**Participants who were given antihypertensive medications by the MoH in the last month**				<0.001
Yes	801 (62.48%)	492(72.67%)	309(51.07%)	
No	380 (29.64%)	165(24.37%)	215(35.54%)	
Never taken medicine	101 (7.88%)	20 (2.95%)	81 (13.39%)	
**Place of delivery of antihypertensive medications**				0.022
Health Post	721 (90.01%)	444(90.24%)	277(89.64%)	
Health Center	40 (4.99%)	18 (3.66%)	22 (7.12%)	
Participant’s house	40 (4.99%)	30 (6.10%)	10 (3.24%)	
**Actions carried out by participants who did not receive medication**				0.002
Did not take medication	162 (42.63%)	62 (37.58%)	100(46.51%)	
Bought the medication	148 (38.95%)	58 (35.15%)	90 (41.86%)	
Had enough medicine for a month or more	48 (12.63%)	31 (18.79%)	17 (7.91%)	
Other	22 (5.79%)	14 (8.48%)	8 (3.72%)	
**Frequency of taking antihypertensive medications**				<0.001
Always	866 (73.33%)	527(80.09%)	339(64.82%)	
Sometimes	208 (17.61%)	88 (13.37%)	120(22.94%)	
Never	107 (9.06%)	43 (6.53%)	64 (12.24%)	

Table 3 presents participants’ reporting of receipt of coaching sessions, use of home blood pressure monitors, and reasons for non-implementation of these two strategies. Of participants in the intervention group, 62% had received at least one health coaching session in the last three months, with Chiquimula being the health area with the highest percentage, 82%. Most of the participants that had received a health coaching session in the last three months, 92%, reported having received it at the health post; however, 40% of participants from Baja Verapaz reported having received it at home. Among the most important reasons for not having received at least one health coaching session in the last three months were: lack of transportation due to COVID-19 restrictions (40%), auxiliary nurses did not want to give the health coaching session (37%), and participant’s lack of time (20%).

**Table 3:** Study activities carried out by participants in the intervention group, June 2020.

	Total	Baja Verapaz	Chiquimula	Huehuetenango	Sololá	Zacapa

**Health coaching sessions**						
**Participants who received at least one session in the last three months**	423(62.48%)	47(55.95%)	162(82.23%)	62(44.60%)	129(60.56%)	23(52.27%)
Place where the participants received the sessions*						
Health Post	390(92.20%)	38(80.85%)	150(92.59%)	53(85.48%)	127(98.45%)	22(95.65%)
Participant’s house	40(9.46%)	19(40.43%)	13(8.02%)	4(6.45%)	3(2.33%)	1(4.35%)
Health Center	1(0.24%)	0(0%)	0(0%)	1(1.61%)	0(0%)	0(0%)
Reasons for not having received the session*						
Lack of transportation due to COVID-19 restrictions	101(39.76%)	14(37.84%)	15(42.86%)	27(35.06%)	33(39.29%)	12(57.14%)
Auxiliary nurses did not want to give the health coaching session	95(37.40%)	2(5.41%)	1(2.86%)	44(57.14%)	47(55.95%)	1(4.76%)
Lack of time	52(20.47%)	7(18.92%)	13(37.14%)	12(15.58%)	15(17.86%)	5(23.81%)
Do not want to leave home due to the risk of COVID-19 infection.	31(12.20%)	2(5.41%)	12(34.29%)	12(15.58%)	5(5.95%)	0(0%)
Forgot the appointments	23(9.06%)	4(10.81%)	1(2.86%)	5(6.49%)	12(14.29%)	1(4.76%)
Do not like the sessions	7(2.76%)	6(17.14%)	0(0%)	0(0%)	1(1.19%)	0(0%)
Lack of money to pay for transportation	3(1.18%)	0(0%)	0(0%)	0(0%)	3(3.57%)	0(0%)
Other	39(15.35%)	13(35.14%)	8(22.86%)	7(9.09%)	3(3.57%)	8(38.10%)
**Blood pressure measurement at home**						
**Measurement at home two days a week, once in the morning and again at night every day**	546(80.65%)	72(85.71%)	157(79.70%)	105(75.54%)	171(80.28%)	41(93.18%)
Reasons not to measure blood pressure as directed*						
The participant forgets to do it	57(43.51%)	4(33.33%)	22(55.00%)	0(0%)	29(69.05%)	2(66.67%)
Do not know how to do it	50(38.17%)	5(41.67%)	7(17.50%)	21(61.76%)	16(38.10%)	1(33.33%)
Lack of time	30(22.90%)	3(25.00%)	9(22.50%)	9(26.47%)	9(21.43%)	0(0%)
Do not like to do it	17(12.98%)	0(0%)	5(12.50%)	4(11.76%)	8(19.05%)	0(0%)
The monitor has no batteries	4(3.05%)	0(0%)	0(0%)	2(5.88%)	2(4.76%)	0(0%)
Other	20(15.27%)	5(41.67%)	11(27.50%)	4(11.76%)	0(0%)	0(0%)

*Participants could answer more than one option.

Regarding the use of home blood pressure monitors, 81% answered that they were using it as instructed – two times a day, at least twice a week – with Zacapa being the health area with the highest percentage (93%). Among the most relevant reasons reported for not having measured their blood pressure at home were: participant forgot that they had to do it (44%), the participant did not know how to do it (38%), and lack of time (23%).

## Discussion

The use of monitoring phone calls was an important complement to our ongoing study in order to capture data about availability of medications, delivery of two of the implementation strategies, and to understand reasons for delivery challenges during the COVID-19 pandemic. There was a significant difference between groups regarding medication availability (73%, 51%) and adherence to treatment (80%, 64%); participants in the control group had comparatively less medication available to them which may have resulted in greater out-of-pocket expense for medications or foregoing treatment. The Ministry of Health’s efforts during the pandemic focused on delivering medications to study participants, which is key to the evidence-based intervention: a protocol-based hypertension treatment [13]. Despite this initiative, MoH personnel were restricted in the delivery of medications, particularly due to there being less staff because auxiliary nurses were mobilized to COVID-19 checkpoints, care facilities, and prevention centers. The results of the phone calls made in June showed that no more than 80% of participants received medication in the last month, although there was a statistically significant difference in the intervention group (73%) vs. control group (51%). At the time of the calls, 86% of intervention group participants reported having enough medication at home for the next month, as compared to 69% in the control group. In both groups, it was noted that most participants received medications at health posts, rather than at home. Although understandable considering that study health posts were understaffed during the health emergency – with only one nurse available in most cases – it was expected to be a higher percentage. Some of the negative consequences for participants who did not receive antihypertensive medications at home included not taking medication during this time or having to buy medications out-of-pocket.

The two other components of the intervention that could be explored through phone calls with intervention group participants were health coaching sessions and home blood pressure monitoring. The first component was strongly affected by changes in the health workforce due to the pandemic, as only 62% of participants received at least one health coaching session in the last three months, considering that this is the minimum number of sessions they should receive if their blood pressure is controlled [13]. Like medication delivery, sessions were mostly (>90%) given at health posts. The most frequent and important reason that participants reported for not having received health coaching sessions during this period was lack of transportation to health posts due to the government-imposed suspension of all types of public transportation that began in March 2020 and continued through August 2020. Concerning home blood pressure measurement, most participants had a high frequency of measurement at home. Of those who did not measure their blood pressure as directed, the most frequent reasons included a lack of knowledge on how to use the digital monitor or forgetting that they had to do it. The correct method of blood pressure measurement was taught and reinforced during health coaching sessions, so the lack of use of digital monitors could be linked to the low number of sessions received.

While our study provided insight into the monitoring of availability of medications and progress on two key implementation strategies (coaching sessions and use of a home blood pressure monitor) during the initial months of the COVID-19 pandemic in Guatemala, this analysis has several important limitations. It was not possible to measure blood pressure control via phone calls, which is a key parameter for the ongoing study; the reason for this is that blood pressure measurement was to be captured using a standardized procedure by trained and certified field data collectors, and patient reporting on self- BP measurements would not be consistent. In addition, more than half of our participants are elderly, are not able to read or write, and usually require assistance to perform blood pressure measurement and recording, so it was not feasible for them to share these values with us through telephone calls.

Cost is always important to consider when making decisions about program implementation. This study did not evaluate the economic burden of the telephone calls made to each participant. In addition, although a high percentage of participants were successfully contacted, this was made through an intense effort by study staff using project resources. This could be a significant limitation for the MoH to implement a phone-call-based monitoring system in the case of a pandemic in the future. Although outside of the scope of this paper, an important factor that needs to be addressed in ongoing study on hypertension control is representativeness: more than 70% of the population is female. One likely reason for lower participation by men is that Guatemala’s workforce in rural areas is largely male and women stay at home and are present during study-related visit times.

This analysis showed that adherence was high overall (and higher in the intervention group), but due to the nature of the non-validated, simplified questionnaire used during phone interviews, a more complete assessment of the type of medications, frequency and dosage was not possible.

Training on how to use the blood pressure monitor is critical as shown in our analysis, where 38% of participants did not measure their BP at home at least two days a week and 19% reported they did not know how to. Coaching sessions are of high importance for patient training and re-training for correct BP measurement at home. Home blood pressure monitoring has been extensively studied, and its measurements can even be used as guidance for blood pressure treatment decisions. These home measurements may even have superior prognostic value compared to in-office blood pressure measurements and evidence suggests that home monitoring improves long-term hypertension control rates. Some guidelines recommend home monitoring for controlled and not-controlled hypertensive patient management [15]. While this method has many benefits, it also has several limitations: many blood pressure monitors available on the market are not validated, and thus may be inaccurate; its use requires comprehension and training for more accurate measurements and acquiring a home blood pressure monitor is costly (which in a LMIC such as Guatemala, is a key barrier for most of the country’s population) [16].

This analysis has implications for hypertension care and implementation research in Guatemala and other low-resource settings during a public health emergency. Our results suggest that care for hypertensive patients may have been delayed or paused, both because of changing MoH priorities and staffing patterns, and because of patients’ reduced ability and confidence to visit health centers and health posts. The use of telephone calls to monitor patients and implementation progress can provide valuable information on the extent of intervention delivery. In addition, we saw that it was possible to speak with most study participants in rural settings via cellular phone when it was not an option to see them in person. The current COVID-19 pandemic has made traditional healthcare challenging for some of the most vulnerable populations. While it is an unprecedented situation, public health systems and research teams must be prepared to adapt amid public health emergencies.

## Conclusion

In rural settings in LMIC such as Guatemala, access to antihypertensive drugs and health services during the pandemic has been impaired, with medication delivery being less than expected and a decreased number of health coaching sessions given, even after accounting for the program’s implementation, mostly due to restricted public transportation on top of the decreased availability of health personnel. This is understandable, since health personnel focused on COVID-19 matters during this time, so implementation of new programs have been affected. This study showed potential strategies to monitor and follow-up patients at home for hypertension control.

During public health emergencies, this study showed that traditional care for hypertensive patients could be delayed, or even paused, due to shifting MoH priorities, staffing patterns and patients’ ability to visit health posts. We can safely assume that during these public health emergencies, phone calls can provide substantial information to monitor hypertension care and implementation research.

## Data Accessibility Statement

The datasets generated during and/or analyzed during the current study are available from the corresponding author on reasonable request.

## Additional Files

The additional files for this article can be found as follows:

10.5334/gh.954.s1 Questionnaire. Participants monitoring through phone calls.

10.5334/gh.954.s2 Script of telephone calls. Informative Dialogue for Phone call.
