# Supplementary material for: Monitoring Study Participants and Implementation with Phone Calls to Support Hypertension Control During the COVID-19 Pandemic: The Case of a Multicomponent Intervention Trial in Guatemala
Source: Glob Heart. 2021 Nov 24;16(1):77. doi: 10.5334/gh.954 (PMC8622336; doi:10.5334/gh.954)
Supplement: Questionnaire. — Participants monitoring through phone calls. [file gh-16-1-954-s1.pdf]

Participants monitoring through phone calls

Participants ID ---

Health Area

☐Baja Verapaz

☐Chiquimula

☐Zacapa

☐Huehuetenango

☐Solola

Baja Verapaz Health district

☐Rabinal

☐Cubulco

☐Salama

☐San Miguel Chicaj

Chiquimula health district

☐Chiquimula

☐Camotan

☐Jocotan

☐Quetzaltepeque

☐Esquipulas

☐Ipala

☐Olopa

☐Concepcion Las Minas

☐San Juan Ermita

☐San Jacinto

Zacapa health district

☐La union

☐Gualan

Huehuetenango health district

☐Huehuetenango Sur

☐Huehuetenango Norte

☐Cuilco

☐La Libertad

☐Ixtahuacan

☐Chiantla

☐Santa Barbara

☐Todos Santos Cuchumatan

☐Colotenango

☐San Sebastian Huehuetenango

Solola health district

☐Solola

☐Panajachel

☐San Lucas Toliman

☐Santiago Atitlan

☐Santa Lucia Utatlan

☐Nahuala

☐San Pablo La Laguna

☐San Pedro La Laguna

☐Xejuyup

☐Guineales

Medications

Read : Now, I would like to ask you some questions regarding your high blood pressure and your medications.

1. **Did you receive hypertensive medication during the last month from the ministry of health personnel?**

   ☐ yes.
   ☐ No.
   ☐ I have never taken medication/ Never has been prescribed medications

2. **Where was your high blood pressure medication given last month?**

   ☐ Health post
   ☐ Health center
   ☐ Participant´s home

3. **What did you do when the medication was not given to you last month?**

   ☐ Bought medicine with his/her own money.
   ☐ had enough medication for a month or more.
   ☐ Received medicines as a gift or donation.
   ☐ Borrowed medication from some family member, friend, or neighbor.
   ☐ did not take any medication during that period.
   ☐ receives medication from IGSS.
   ☐ Another: __________________

4. **How often did you take your medication for high blood pressure last month?**

   ☐ Always
   ☐ sometimes
   ☐ Never

☐ Intervention group → Continue next section
☐ No →End questionnaire.

Health coaching sessions.

Read: Now, I am going to ask you about the health coaching sessions you had during the program. A coaching session is a meeting at the health post or your home, where the auxiliary nurse explains to you and a family member, what hypertension is, how to use your blood pressure monitor, what should you eat, how much exercise to do and how to improve control of your blood pressure with medication.

5. **During the last three months since March 16th, have you had any health coaching sessions?**

☐ Yes
☐ No

6. **how many coaching sessions did you have during the last 3 months?**

☐ 1
☐ 2
☐ 3

7. **¿ where did you have your coaching sessions**

Select all that apply

☐ participant´s home.
☐ family member or friend´s home
☐ Health post
☐ Health center
☐ another place inside the community
☐ Another: __________________

8. **If you have not received any coaching session, what was the reason?**
. *Note. This is an open question. Let the participant answer, then select all the options that apply, o document any other barriers*

☐ lack of time
☐ lack of transportation due to covid19 restrictions
☐ does not want to leave the house for risk of Covid 19 contagion.
☐ lack of money for transportation
☐ forgets about the appointments
☐ doesn't like the health coaching sessions
☐ healthcare personnel did not want to give the sessions
☐ Another: ____

9. **During the last month. Have you measured your blood pressure at home twice a week, once in the morning and another at night?**

☐ Yes
☐ No

1. **If you have not measured your blood pressure, what is the reason?**

☐ lack of time
☐ forgets to do it
☐ does not know how to do it
☐ does not like to do it
☐ Blood pressure monitor does not have batteries
☐ blood pressure monitor misplaced or stolen
☐ another: ________________________

Observations ____

Evaluator initials: ☐☐

Evaluator code: ☐☐

Date the form is entered: ☐☐/☐☐/☐☐☐☐
