# Supplementary material for: Monitoring Study Participants and Implementation with Phone Calls to Support Hypertension Control During the COVID-19 Pandemic: The Case of a Multicomponent Intervention Trial in Guatemala
Source: Glob Heart. 2021 Nov 24;16(1):77. doi: 10.5334/gh.954 (PMC8622336; doi:10.5334/gh.954)
Supplement: Script of telephone calls. — Informative Dialogue for Phone call. [file gh-16-1-954-s2.pdf]

**Informative Dialogue for Phone call**<br>**INTERVENTION DISTRICTS**	
**Aspects that must be spoken with participant**	**Information**
**1. General information and RedCap questionnaire completion**	Good day, this is **XXXXX**, field evaluator from INCAP. I have been communicating with you about INCAP's program for hypertension control.<br>During this call, I would like to make you some questions about you and your health. Do you have some time available for me to ask you these questions and talk briefly about your health and this program's activities?<br>**FOR THE EVALUATOR:** Remember that this phone call will approximately last 15 minutes. If the participant is busy during this time, the phone call should be scheduled according to the participant's availability. If the participants DOES have time availability at this moment, continue with the next dialogue:<br><br>Due to current national situation, some of the program's activities have been suspended. Still, your blood pressure control is of great importance, so I would like to make you some questions regarding blood pressure medicines you currently take and other activities from this program. The information that you provide during this phone call is confidential. If you desire to stop this call in any moment, you are free to do so. Do you agree for us to ask you some questions? Do you have a question? Can we begin?<br><br>**FOR THE EVALUATOR:** Proceed to ask RedCap's questions.
**2. Treatment adherence encouragement and study's activities compliance (counseling sessions and home blood pressure monitoring)**	**FOR THE EVALUATOR:** After completing RedCap's questionnaire, proceed to read the next dialogue. If at any time the participant asks any type of question about how to take the medication, adverse events, or any other type of medical consult, explain that you are not qualified to give this information, and suggest that they consult a health provider. Besides this, you should immediately inform your supervisor.<br><br>Thank you very much for answering my questions. Now, I would like to talk with you about some of the program's activities that you can continue from home.<br><br>1. **The most important activity that you must continue is to take your blood pressure pills daily.** Remember that increased blood pressure **is a chronic disease; this means that it has no cure. The good news is that it can be controlled through the medication that was prescribed to you.** It is of the uttermost importance that you **take these medicines every day, in the prescribed dosage and schedule** by your doctor or professional nurse**.**<br>If you do not remember which medicine, you should be taking for your blood pressure, at what time, what

	quantity, or have been feeling any type of disturbance every time you take it, you must go to your nearest health center/post and consult a health provider that can help you. Remember that increased blood pressure may not always cause disturbances or symptoms at all. It is still of great importance that you take your medication every day.<br><br>2. **The second activity that you should continue from home is blood pressure monitoring,** with the electronic device that I gave you. **This blood pressure monitoring should be 2 days a week** (Tuesdays and Thursdays for example but can be any 2 days of your preference). On these 2 days of your selection, you should take your blood pressure **one time in the morning and one time in the afternoon. The number that your monitor shows should be annotated on your blood pressure card**. If you do not know how to write or read, someone from your family may help you write down this information.<br>If you or the person that is helping you see that the blood pressure **number on your electronic monitor are equal or greater than 180 mmHg from your systolic blood pressure (number in the top part of your monitor), or equal or greater than 120 mmHg from your diastolic blood pressure (number on the lower part of your monitor), you must consult immediately to a health service**, since you can suffer from a very high blood pressure complication. In addition, if you present intense headache, chest pain, sudden blurred vision, seizures, or fainting, **you must immediately consult to a health service, so a doctor or professional nurse evaluates you.**<br><br>3. **The third activity that you should continue are counseling sessions.** These are talks that you have with the auxiliary nurse at the health post (or at home, in some cases), where you discuss about blood pressure medicine, healthy diet, and exercise.<br><br>**FOR THE EVALUATOR:** In this part, the evaluator must explain to the participant how the corresponding health post personnel will give counseling sessions. Before this call, the evaluator must communicate to the health providers and ask about how the counseling sessions are going to be given, at what times, and where.
**3. Diet and physical activity recommendations**	Lastly, I would like to mention you some actions that will help you be healthier:<br><br>• Consume from 4 to 5 vegetable and fruit portions per day.<br>• Consume less than 1 spoonful of salt per day.

	• Reduce your consumption of grease-rich, sugar-rich, or salt-rich food. This includes soda, junk food, candy, fried food (like fried chicken or fries), canned fruit, or jelly.<br>• Take at least eight daily glasses of water.<br>• Do at least 30 minutes of moderate physical activity, at least 5 days a week.<br>If you plan to change your diet or if you want to start doing physical activities, **you can consult the flip chart that the auxiliary nurse gave you during your first consult to the health post, or you can consult the health post of your community**.
**4. Gratitude and farewell**	Thank you very much for the time you took to take my call today. If you have any question about the program's activities, please communicate with me to the telephone number: ________ (include here the corresponding INCAP evaluator telephone number). Have a nice day.

**Informative Dialogue for Phone call**<br>**CONTROL DISTRICTS**	
**Aspects that must be spoken with participant**	**Information**
**1. General information and RedCap questionnaire completion**	Good day, this is XXXXX, field evaluator from INCAP. I have been communicating with you about INCAP's program for hypertension control.<br>During this call, I would like to make you some questions about you and your health. Do you have some time available for me to ask you these questions and talk briefly about your health and this program's activities?

	**FOR THE EVALUATOR:** Remember that this phone call will approximately last 7 minutes. If the participant is busy during this time, the phone call should be scheduled according to the participant's availability. If the participants DOES have time availability at this moment, continue with the next dialogue:<br><br>Due to current national situation, some of the program's activities have been suspended. Still, your blood pressure control is of great importance, so I would like to make you some questions regarding blood pressure medicines you currently take. The information that you provide during this phone call is confidential. If you desire to stop this call in any moment, you are free to do so. Do you agree for us to ask you some questions? Do you have a question? Can we begin?<br><br>Remember to review the informed consent before making this call, to tell the participants again about the benefits of their participation in this study.<br><br>**FOR THE EVALUATOR:** Proceed to ask RedCap's questions.<br>Make sure to fill only the information pertaining to pharmacological treatment delivery and adherence. Other sections are only filled with intervention districts.
**2. Gratitude and farewell**	**FOR THE EVALUATOR:** If at any time the participant asks any type of question about how to take the medication, adverse events, or any other type of medical consult, explain that you are not qualified to give this information, and suggest that they consult a health provider. Besides this, you should immediately inform your supervisor.<br><br>Before this phone call, the evaluator must inquire how health providers are currently conducting attention at the different health services (attention hours, attention days, specific days for hypertensive persons, medication delivery available to patient's relative or only to patient, home visits, etc.).<br><br>Before this phone call ends, read the next paragraph:<br><br>Thank you very much for the time you took to take my call today. If you have any question about the program's activities, please communicate with me to the telephone number: _______________ (include here the corresponding INCAP evaluator telephone number). Have a nice day.
